# Crystal structures and Hirshfeld surface analyses of *N*,*N*-di­methyl­acetamide–1-(dimethyl-λ^4^-aza­nyl­idene)ethan-1-ol tribromide (1/1), *N*,*N*-di­methyl­acetamide–1-(dimethyl-λ^4^-aza­nyl­idene)ethan-1-ol di­bromido­iodate (1/1) and *N*,*N*-di­methyl­acetamide–1-(dimethyl-λ^4^-aza­nyl­idene)ethan-1-ol di­chlorido­iodate (1/1)

**DOI:** 10.1107/S2056989023005509

**Published:** 2023-07-04

**Authors:** Gunay Z. Mammadova, Dmitriy F. Mertsalov, Dmitriy M. Shchevnikov, Mikhail S. Grigoriev, Mehmet Akkurt, Sema Öztürk Yıldırım, Ajaya Bhattarai

**Affiliations:** a Organic Chemistry Department, Baku State University, Z. Xalilov Str. 23, Az 1148 Baku, Azerbaijan; b Peoples’ Friendship University of Russia (RUDN University), 6 Miklukho-Maklaya St., Moscow 117198, Russian Federation; c Frumkin Institute of Physical Chemistry and Electrochemistry, Russian Academy of Sciences, Leninsky pr. 31, bld. 4, Moscow 119071, Russian Federation; d Department of Physics, Faculty of Sciences, Erciyes University, 38039 Kayseri, Türkiye; e Department of Physics, Faculty of Science, Eskisehir Technical University, Yunus Emre Campus, 26470 Eskisehir, Türkiye; f Department of Chemistry, M.M.A.M.C., Tribhuvan University, Biratnagar, Nepal; Universität Greifswald, Germany

**Keywords:** crystal structure, di­methyl­acetamide, trihalide, hydrogen bond, Hirshfeld surface analysis

## Abstract

In the title compounds, *N*,*N*-di­methyl­acetamide–1-(dimethyl-λ^4^-aza­nyl­idene)ethan-1-ol tribromide (1/1), C_4_H_9_NO·C_4_H_10_NO^+^·Br_3_^−^ or [(C_4_H_9_NO)·(C_4_H_10_NO)](Br_3_), (**I**), *N*,*N*-di­methyl­acetamide–1-(dimethyl-λ^4^-aza­nyl­idene)ethan-1-ol di­bromido­iodate (1/1), C_4_H_9_NO·C_4_H_10_NO^+^·Br_2_I^−^ or [(C_4_H_9_NO)·(C_4_H_10_NO)](Br_2_I), (**II**), and *N*,*N*-di­methyl­acetamide–1-(dimethyl-λ^4^-aza­nyl­idene)ethan-1-ol di­chlorido­iodate (1/1), C_4_H_9_NO·C_4_H_10_NO^+^·Cl_2_I^−^ or [(C_4_H_9_NO)·(C_4_H_10_NO)]·(Cl_2_I), (**III**), all the anions are almost linear in geometry and all the cations, except for the methyl H atoms, are essentially planar. In the crystal structure of (**I**), the cations are linked by pairs of C—H⋯O hydrogen bonds, forming inversion dimers with an *R*_2_^2^(8) ring motif. These dimers also exhibit O—H⋯O hydrogen bonding. Dimerized cation pairs and anions are arranged in columns along the *a* axis. In the crystal of (**II**), the cations are linked by pairs of O—H⋯O and C—H⋯O hydrogen bonds, forming an *R*_4_^4^(14) ring motif. These groups of cations and the anions form individual columns along the *a* axis and jointly reside in planes roughly parallel to (011). In the crystal of (**III**), cations and anions also form columns parallel to the *a* axis, resulting in layers parallel to the (020) plane. Furthermore, the crystal structures of (**I**), (**II**) and (**III)** are consolidated by strong halogen (Br and/or I and/or Cl)⋯H and weak van der Waals inter­actions. In addition to the structural evaluation, a Hirshfeld surface analysis was carried out.

## Abstract 2

In all three title crystals, the cations are linked by O—H⋯O and/or C—H⋯O hydrogen bonds. The three-dimensional packing is further consolidated by strong halogen–hydrogen and weak van der Waals inter­actions.

## 1. Chemical context

Halogenation is a chemical reaction that involves the introduction of one or more halogen atoms to an organic compound. Usually, either direct replacement of hydrogen by a halogen atom or addition of a halogen mol­ecule to double and triple bonds are used. The pathway and stereochemistry of halogenation reactions are strongly dependent on the halogenating agent. However, halogens and inter­halogens are very harmful to health. An effective source of active halogen should be a safe solid substance well soluble in different solvents, with a low pressure of halogen vapour and high content of the active halogen. As a source of halogens, mol­ecular complexes with N- and O-nucleophiles are widely used. However, the N-halogen succinimides slowly decompose when stored and are poorly soluble in some solvents, while the mol­ecular complexes of halogens with N- and O-nucleophiles (for instance, dioxane dibromide or complexes with pyridine) are short-lived (Abdell-Wahab *et al.*, 1957 ▸; Horner *et al.*, 1959 ▸; Zaugg *et al.*, 1954 ▸; Buckles *et al.*, 1957 ▸; Ramachandrappa *et al.*, 1998 ▸; Groebel *et al.*, 1960 ▸; Mohamed Farook *et al.*, 2006 ▸; Sui *et al.*, 2006 ▸). In this context, we synthesized inexpensive and readily available bis­(*N*,*N*-di­methyl­acetamide) hydrogen tri­halides as halogenation agents and source of positively charged halogen ions (Rodygin *et al.*, 1992 ▸; Prokop’eva *et al.*, 2008 ▸). The amide complexes with halogens are excellent reagents for the functionalization of phenols and anilines (Rodygin *et al.*, 1992 ▸; Mikhailov *et al.*, 1993 ▸; Safavora *et al.*, 2019 ▸). They are also used in the synthesis of mono-halogen-substituted ketones (Rodygin *et al.*, 1994*a* ▸; Burakov *et al.*, 2001 ▸; Abdelhamid *et al.*, 2011 ▸; Khalilov *et al.*, 2021 ▸) and the halogenation of various alkenes, alkynes (Rodygin *et al.*, 1994*b* ▸) and bridged ep­oxy-isoindolones (Zaytsev *et al.*, 2017 ▸; Zubkov *et al.*, 2018 ▸; Mertsalov *et al.*, 2021*a* ▸,*b* ▸). The most famous amide complex, *i.e.* Povidone-iodine (PVP-I), also known as iodo­povidone, is an anti­septic used for skin disinfection before and after surgery (Stuart *et al.*, 2009 ▸). Moreover, noncovalent inter­actions play critical roles in synthesis and catalysis, as well as in forming supra­molecular structures due to their significant contribution to the self-assembly process (Gurbanov *et al.*, 2020*a* ▸,*b* ▸, 2022*a* ▸,*b* ▸; Ma *et al.*, 2017 ▸, 2021 ▸; Mahmoudi *et al.*, 2017*a* ▸,*b* ▸; Mahmudov *et al.*, 2011 ▸, 2022 ▸). Similar to hydrogen bonding, the halogen bond has also been used in the design of materials (Shikhaliyev *et al.*, 2019 ▸). We, thus, analyzed such expected respective inter­molecular inter­actions in the isolated and structurally characterized three title aggregates in the context of the present study. 
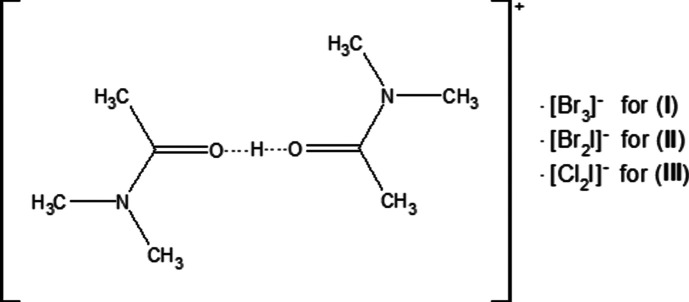


## 2. Structural commentary

**Figure 1:**
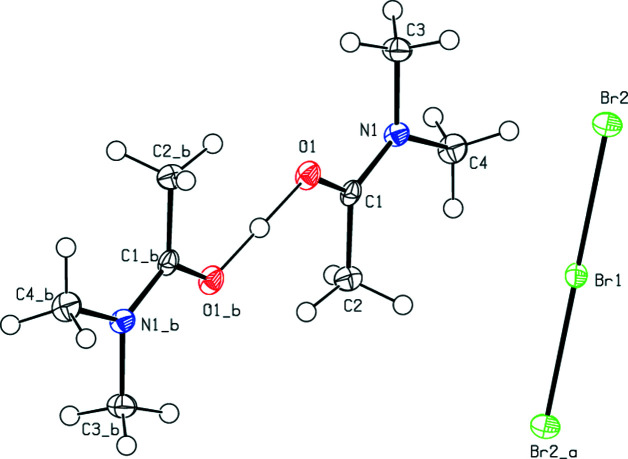
The mol­ecular structure of (**I**), with displacement ellipsoids for the non-H atoms drawn at the 50% probability level.

In the title compounds (**I**), (**II**) and (**III**) (Figs. 1 ▸, 2 ▸ and 3 ▸), the Br_3_^−^, Br_2_I^−^ and Cl_2_I^−^ anions are almost or perfectly linear in geometry. For (**I**), Br1 resides in the centre of inversion symmetry [Br2—Br1—Br2(−*x* + 1, −*y* + 1, −*z* + 1) = 180.0°], with Br1—Br2 distances of 2.53725 (17) Å. The cations, except for their methyl H atoms, are essentially planar [r.m.s. deviation = 0.041 (1) Å for O1]. For (**II**), the angles and distances of the anion are Br1—I1—Br2 = 177.942 (5)°, I1—Br1 = 2.7244 (2) Å and I1—Br2 = 2.68597 (19) Å. These values are in agreement with data reported in the literature (Gardberg *et al.*, 2002 ▸). The cations, except for their methyl H atoms, are again essentially planar [r.m.s. deviations = −0.018 (1) Å for O1 and −0.038 (2) Å for C7]. For (**III**), I1 resides in the centre of inversion symmetry [Cl1—I1—Cl1(−*x* + 1, −*y* + 1, −*z* + 1) = 180.0°], with distances of I1—Cl1 = 2.53973 (18) Å. The cations, except for their methyl H atoms, are planar and all reside on mirror planes.

In (**I**), (**II**) and (**III**), the O—C and N—C bond distances of the cation all fall between single and double bond values, with C1—N1 = 1.3134 (17) Å and C1—O1 = 1.2786 (16) Å for (**I**), C1—N1 = 1.3168 (16) Å, C5—N2 = 1.3121 (16) Å, C1—O1 = 1.2771 (15) Å and C5—O2 = 1.2794 (15) Å for (**II**), and C1—N1 = 1.3161 (8) Å and C1—O1 = 1.2750 (8) Å for (**III**). The corresponding bond lengths of the three compounds are in good agreement with each other and with the literature.

## 3. Supra­molecular features and Hirshfeld surface analysis

**Figure 4:**
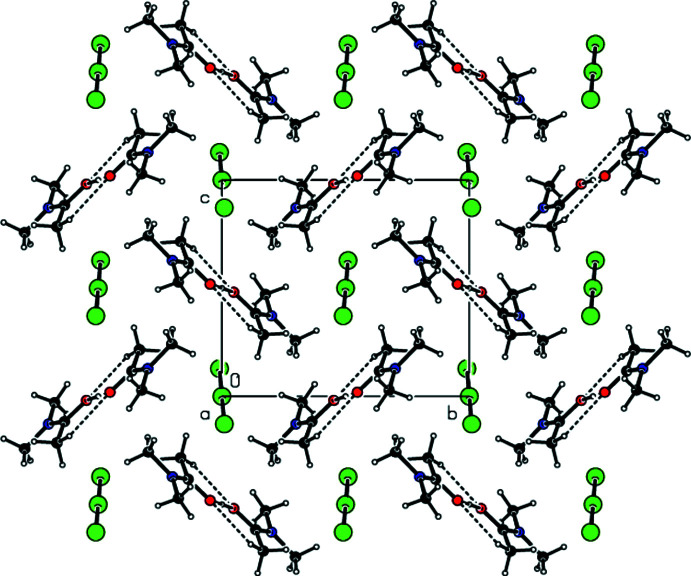
A view along the *a* axis of the O—H⋯O and C—H⋯O inter­actions in the crystal structure of (I).

**Figure 6:**
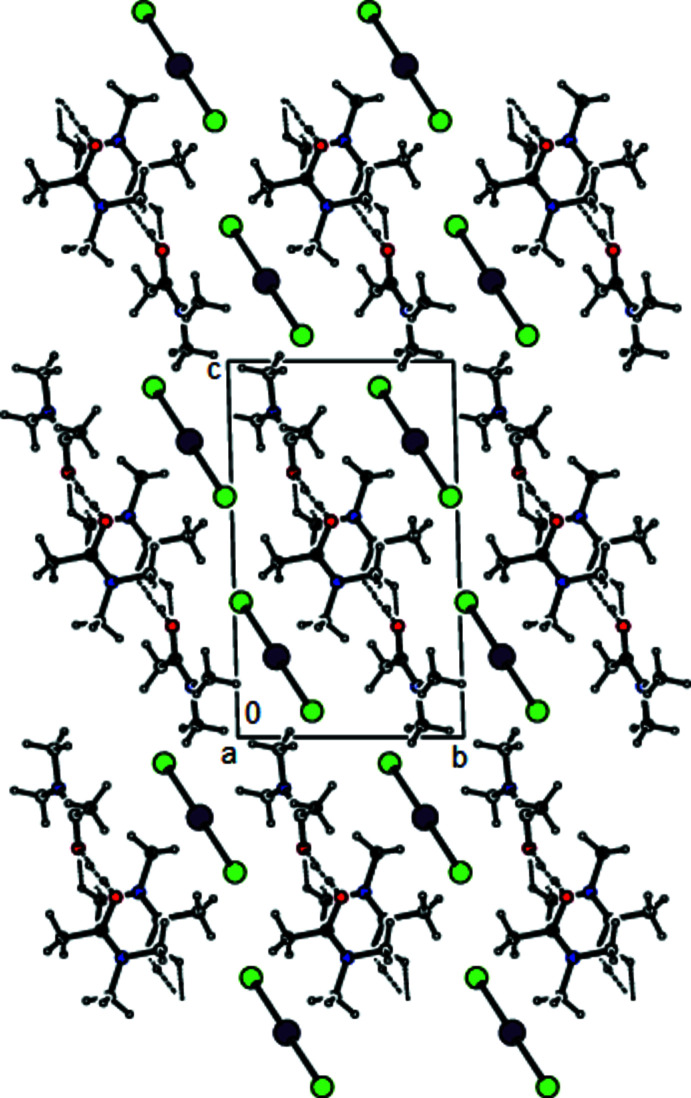
A view along the *a* axis of the O—H⋯O and C—H⋯O inter­actions in the crystal structure of (II).

**Figure 8:**
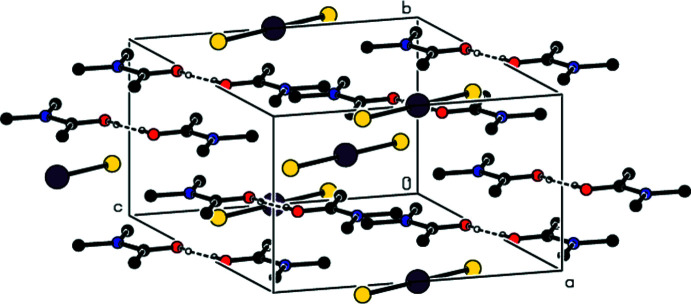
A view along the *a* axis of the O—H⋯O inter­actions in the crystal structure of (III).

In the crystal of (**I**), the cations are linked by pairs of C—H⋯O hydrogen bonds (symmetry code: −*x* + 2, −*y* + 1, −*z* + 2), forming inversion dimers with an 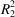 (8) ring motif (Bernstein *et al.*, 1995 ▸) (Table 1 ▸ and Fig. 4 ▸). These dimers also exhibit O—H⋯O hydrogen bonds (symmetry code: −*x* + 2, −*y* + 1, −*z* + 2). Dimerized cation pairs and anions are arranged in columns along the *a* axis (Figs. 4 ▸ and 5 ▸). In the crystal of (**II**), two cations are refined in the asymmetric unit. These cations are linked by pairs of O—H⋯O and C—H⋯O hydrogen bonds, forming an 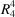 (14) ring motif (Table 2 ▸, and Figs. 6 ▸ and 7 ▸). The groups of cations and anions form columns along the *a* axis and reside in planes parallel to (011) (Figs. 6 ▸ and 7 ▸). In the crystal of (**III**), cations and anions are arranged in columns parallel to the *a* axis, forming layers parallel to the (020) plane (Table 3 ▸, and Figs. 8 ▸ and 9 ▸). Furthermore, the crystal structures of (**I**), (**II**) and (**III)** are consolidated by strong halogen (Br and/or I and/or Cl)⋯H bonding inter­actions, Coulombic attraction and weak van der Waals inter­actions (Tables 4 ▸ and 5 ▸) between the cations and anions in three dimensions.

The O⋯O distances in (**I**), (**II**) and (**III**) are 2.4224 (15), 2.4278 (14) and 2.4261 (9) Å, respectivly, and are thereby within the range (2.31–2.63 Å) found for short/strong classical hydrogen bonds (Hussain & Schlemper, 1980 ▸; Behmel *et al.*, 1981 ▸).

**Figure 10:**
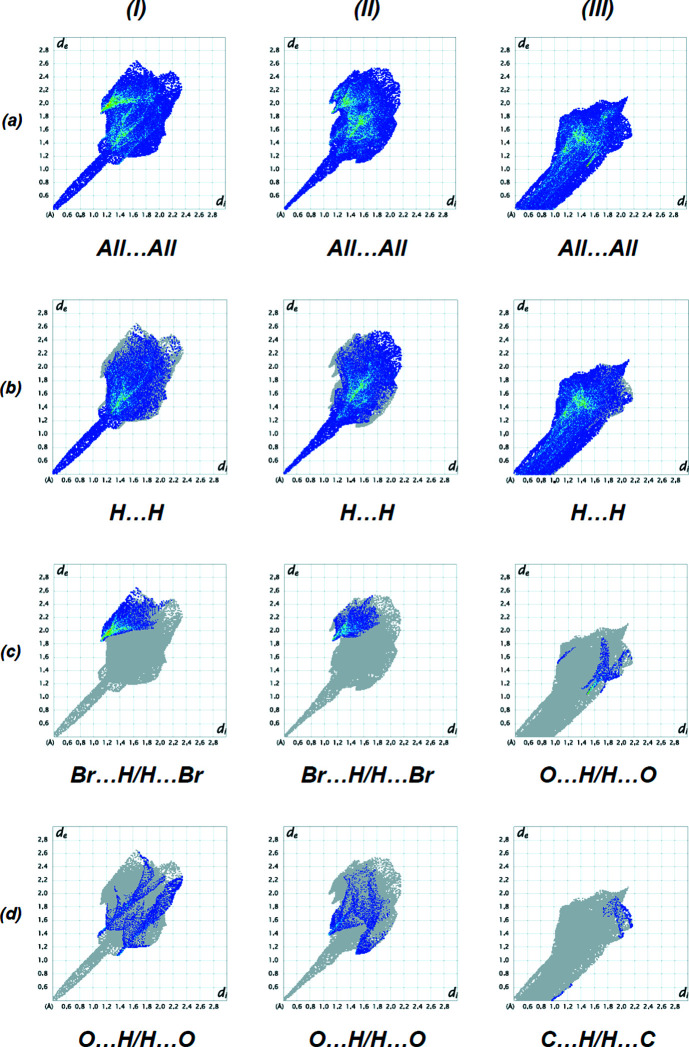
A view of the two-dimensional fingerprint plots for compounds (**I**), (**II**) and (**III**), showing (*a*) all inter­actions, and separated into (*b*) H⋯H, (*c*) Br⋯H/H⋯Br for (**I**) and (**II**), O⋯H/H⋯O for (**III**) and (*d*) O⋯H/H⋯O for (**I**) and (**II**), C⋯H/H⋯C for (**III**) inter­actions. The *d*_i_ and *d*_e_ values are the closest inter­nal and external distances (in Å) from given points on the Hirshfeld surface contacts.

The Hirshfeld surface analysis and the associated two-dimensional fingerprint plots over the cations of (**I**), (**II**) and (**III**) were carried out and created with *CrystalExplorer17.5* (Spackman *et al.*, 2021 ▸). A summary of the short inter­atomic contacts in (**I**), (**II**) and (**III**) is given in Table 4 ▸. The two-dimensional fingerprint plots for compounds (**I**), (**II**) and (**III**) are shown in Fig. 10 ▸. The principal inter­atomic inter­actions for the title compound [Figs. 10 ▸(*b*)–(*d*) and Table 5 ▸] are delineated into H⋯H [57.5% for (**I**); 60.3% for (**II**); 88.9% for (**III**)], Br⋯H/H⋯Br [24.0% for (**I**); 15.2% for (**II**)], O⋯H/H⋯O [6.5% for (**III**)] and O⋯H/H⋯O [13.3% for (**I**); 12.0% for (**II**)] and C⋯H/H⋯C [2.0% for (**III**)] contacts.

The respective differences in the crystal structures of the three title compounds [(**I**): space group, monoclinic *P*2_1_/*n*, *Z* = 2; (**II**): space group, triclinic *P* 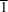 , *Z* = 2; (**III**): space group, monoclinic *C*2/*m*, *Z* = 2], may be the result of small deviations in the inter­actions arising from the different crystal systems and packing, as well as from the variations in the anions of the compounds.

## 4. Database survey

A database search was carried out using *ConQUEST* (Bruno *et al.*, 2002 ▸), part of Version 2022.3.0 of the Cambridge Structural Database (Groom *et al.*, 2016 ▸). A search for structures with the simultaneous presence of *N*,*N*-di­methyl­acetamide and its respective protonated form resulted in ten hits. Two compounds are deposited twice, so there are only eight related structures known. Compounds closely related to the title compound are: bis­[hexa­kis­(*N*,*N*-di­methyl­acetamide-κ*O*)aluminium(III)] bis­(*N*,*N*-di­methyl­acetamide)­ium hepta­kis­(perchlorate) (CSD refcode DEGBOH; Suzuki & Ishiguro, 2006 ▸), hydrogen bis­(*N*,*N*-di­methyl­acetamide) tetra­chloro­gold(III) (HDMAAU; Hussain *et al.*, 1980 ▸), hydrogen bis­(di­methyl­acetamide) tribromide [SEGMOG (Gubin *et al.*, 1988 ▸) and SEGMOG01 (Mikhailov *et al.*, 1992 ▸)].

In the crystal of DEGBOH (space group: monoclinic *P*2_1_ *n*, *Z* = 2), the Al^3+^ ion is surrounded by dma mol­ecules (dma = di­methyl­acetamide) in an octa­hedral arrangement. The dma mol­ecules are essentially planar. Three Al—O—C—N torsion angles [138.8 (8)–149.3 (4)°] are found to deviate significantly from 180°. The centrosymmetric cation has the bridging H atom at the centre of inversion. The planar structure is essentially the same as those reported for [H(dma)_2_]^+^ cations; the O⋯O distance [2.386 (8) Å] is within the range (2.31–2.63 Å) found for short hydrogen bonds (Hussain & Schlemper, 1980 ▸; Behmel *et al.*, 1981 ▸).

In the crystal of HDMAAU (space group: monoclinic *P*2_1_ *a*, *Z* = 2), the structure consists of distinct [AuCl_4_]^−^ anions and [H(dma)_2_]^+^ cations, with the gold and the bridging H atoms located at centres of symmetry. The hydrogen bond is ‘symmetrical’ as a result of crystallographic requirements. The O⋯O distance is 2.430 (16) Å. Thermal motion analysis indicates that methyl groups attached to nitro­gen have higher rotational amplitudes, resulting in short apparent C—H bond lengths [average 0.96 (4) Å] compared with the methyl group attached to a carbonyl C atom which has an average C—H bond length of 1.02 (2) Å.

In the crystal of SEGMOG (space group: monoclinic *P*2_1_ *c*, *Z* = 2), two *N*,*N*-di­methyl­acetamide mol­ecules in the asymmetric unit are connected to each other by an O—H⋯O hydrogen bond, essentially sharing the central H atom. These mol­ecules and the Br—Br—Br groups are arranged in columns parallel to the *a* axis. The arrangement is consolidated in the crystal packing by van der Waals inter­actions between these columns.

In the crystal of SEGMOG01 (space group: monoclinic *P*2_1_ *n*, *Z* = 2), the unit-cell parameters and the arrangement of the mol­ecules are relatively similar to the older structure (SEGMOG), while the H atom bridging the the two acetamides was not refined.

## 5. Synthesis and crystallization

### 5.1. General procedure

To a solution of di­methyl­acetamide (9.28 ml, 0.1 mol) in 0.09 mol of 38% hydro­chloric or 40% hydro­bromic acid under stirring and cooling in an ice–water bath, 0.05 mol iodine monochloride (8.10 g, 0.05 mol), iodine monobromide (10.35 g, 0.05 mol) or bromine (4.00 g, 0.05 mol) was added gradually. The mixture was stirred for 1 h and the crystals were filtered off, dried and recrystallized from methanol to give the target bis­(*N*,*N*-di­methyl­acetamide) hydrogen halides as orange colored solids. Single crystals of bis­(*N*,*N*-di­methyl­acetamide) hydrogen halides were obtained by slow crystallization from methanol.

### 5.2. *N*,*N*-Di­methyl­acetamide–1-(dimethyl-λ^4^-aza­nyl­idene)ethan-1-ol tribromide (1/1), (I)

Bright orange crystals (Rodygin *et al.*, 1992 ▸; Gubin *et al.*, 1988 ▸), yield 81% (16.8 g), m.p. 361–362 K. IR (KBr), ν (cm^−1^): 1664 (NCO). ^1^H NMR (700.2 MHz, CDCl_3_): δ (*J*, Hz) 12.51 (*br s*, 1H), 3.28 (*s*, 3H, NCH_3_), 3.19 (*s*, 3H, NCH_3_), 2.45 (*s*, 3H, CH_3_); ^13^C{^1^H} NMR (176 MHz, CDCl_3_): δ 174.5, 39.7, 37.5, 19.9.

### 5.3. *N*,*N*-Di­methyl­acetamide–1-(dimethyl-λ^4^-aza­nyl­idene)ethan-1-ol di­bromido­iodate (1/1), (II)

Bright-orange crystals, yield 44% (10.2 g), m.p. 343–344 K. IR (KBr), ν (cm^−1^): 1606 (NCO). ^1^H NMR (700.2 MHz, CDCl_3_): δ (*J*, Hz) 10.72 (*br s*, 1H), 3.28 (*s*, 3H, NCH_3_), 3.19 (*s*, 3H, NCH_3_), 2.46 (*s*, 3H, CH_3_); ^13^C{^1^H} NMR (176 MHz, CDCl_3_): δ 174.6, 39.6, 37.5, 20.1.

### 5.4. *N*,*N*-Di­methyl­acetamide–1-(dimethyl-λ^4^-aza­nyl­idene)ethan-1-ol di­chlorido­iodate (1/1), (III)

Bright orange crystals, yield 75% (14 g), m.p. 364–365 K. IR (KBr), ν (cm^−1^): 1611 (NCO). ^1^H NMR (700.2 MHz, CDCl_3_): δ (*J*, Hz) 9.98 (*br s*, 1H), 3.25 (*s*, 3H, NCH_3_), 3.17 (*s*, 3H, NCH_3_), 2.41 (*s*, 3H, CH_3_); ^13^C{^1^H} NMR (176 MHz, CDCl_3_): δ 174.2, 39.4, 37.2, 19.8.

## 6. Refinement

Crystal data, data collection and structure refinement details are summarized in Table 6 ▸. In compounds (**I**), (**II**) and (**III**), the C-bound H atoms were positioned geometrically, with C—H = 0.98 Å (for methyl H atoms), and constrained to ride on their parent atoms, with *U*_iso_(H) = 1.5*U*_eq_(C). The hy­droxy H atoms were found in the difference Fourier maps and their coordinates were refined freely, with *U*_iso_(H) = 1.5*U*_eq_(O). In (**I**), the H atom of the OH group is located in a special position (1.0, 0.5, 1.0) with an occupancy of 0.5 for the rrefined atom. In (**II**), the H atoms of the OH groups are disordered over two positions, with occupancies of 0.49 and 0.51. In (**III**), the H atom of the OH group was refined with an occupancy of 0.25 for its position close to an inversion centre in between the O atoms of two acetamides and simultaneously residing on a mirror plane.

## Supplementary Material

Crystal structure: contains datablock(s) I, II, III, global. DOI: 10.1107/S2056989023005509/yz2034sup1.cif

Structure factors: contains datablock(s) I. DOI: 10.1107/S2056989023005509/yz2034Isup2.hkl

Structure factors: contains datablock(s) II. DOI: 10.1107/S2056989023005509/yz2034IIsup3.hkl

Structure factors: contains datablock(s) III. DOI: 10.1107/S2056989023005509/yz2034IIIsup4.hkl

CCDC references: 2271693, 2271692, 2271691

Additional supporting information:  crystallographic information; 3D view; checkCIF report

## Figures and Tables

**Figure 2:**
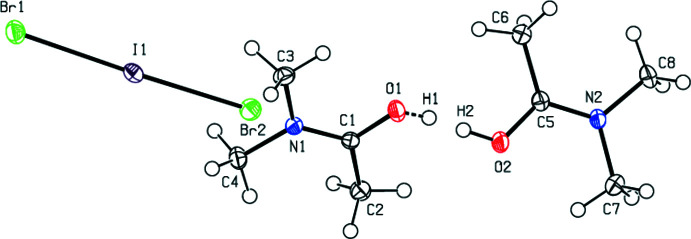
The mol­ecular structure of (**II**), with displacement ellipsoids for the non-H atoms drawn at the 50% probability level. Symmetry codes: (_a) −*x* + 1, −*y* + 1, −*z* + 1; (_b) −*x* + 2, −*y* + 1, −*z* + 2.

**Figure 3:**
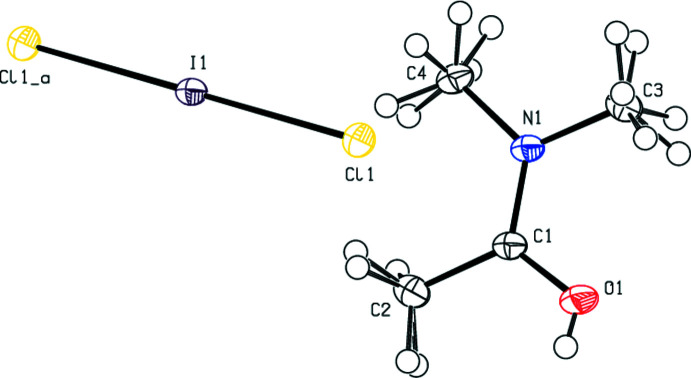
The mol­ecular structure of (**III**), with displacement ellipsoids for the non-H atoms drawn at the 50% probability level. Symmetry code: (_a) −*x* + 1, *y*, −*z* + 1.

**Figure 5:**
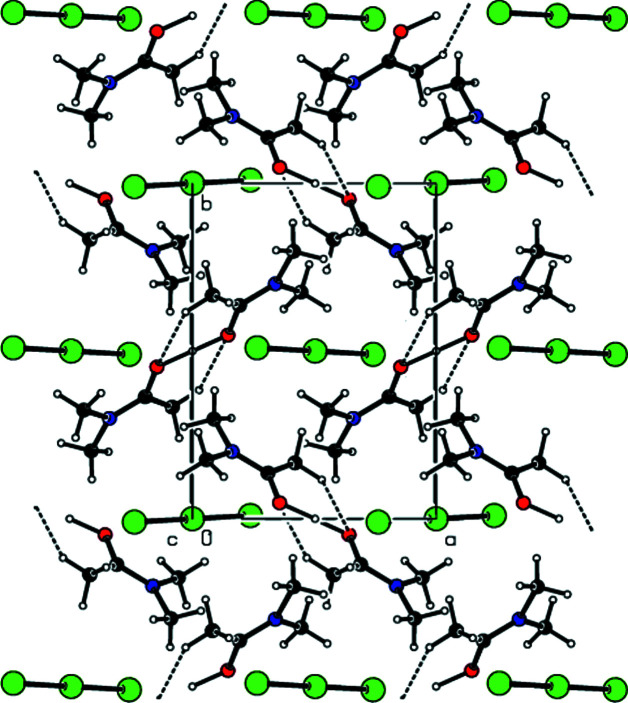
A view along the *c* axis of the O—H⋯O and C—H⋯O inter­actions in the crystal structure of (I).

**Figure 7:**
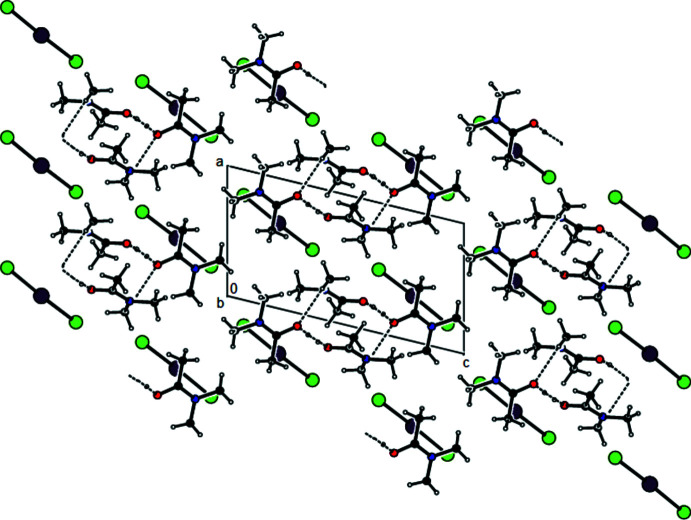
A view along the *b* axis of the O—H⋯O and C—H⋯O inter­actions in the crystal structure of (II).

**Figure 9:**
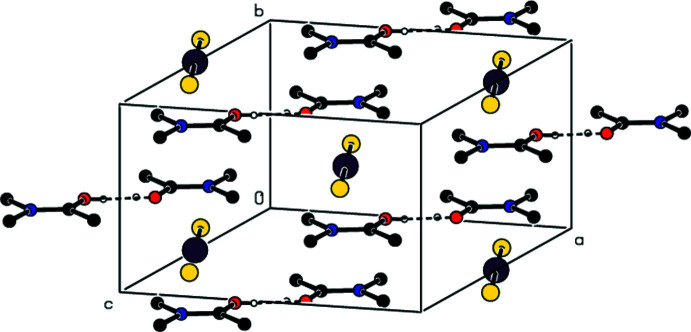
A view along the *c* axis of the O—H⋯O inter­actions in the crystal structure of (III).

**Table 1:** Hydrogen-bond geometry (Å, °) for (I)

*D*—H⋯*A*	*D*—H	H⋯*A*	*D*⋯*A*	*D*—H⋯*A*
C2—H2*A*⋯O1^i^	0.98	2.52	3.2622 (18)	132
C2—H2*B*⋯Br2^ii^	0.98	3.14	4.0788 (15)	162
C2—H2*C*⋯Br1^iii^	0.98	3.13	3.9596 (14)	143
C3—H3*A*⋯Br2^iv^	0.98	3.10	4.0216 (15)	158
C3—H3*C*⋯Br2	0.98	3.05	3.8847 (15)	143
O1—H1⋯O1^i^	1.21	1.21	2.4224 (15)	180

Symmetry codes: (i) 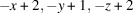 ; (ii) 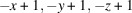 ; (iii) 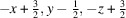 ; (iv) 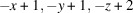 .

**Table 2:** Hydrogen-bond geometry (Å, °) for (II)

*D*—H⋯*A*	*D*—H	H⋯*A*	*D*⋯*A*	*D*—H⋯*A*
O1—H1⋯O2	0.75 (5)	1.69 (5)	2.4278 (13)	173 (4)
O2—H2⋯O1	0.85 (5)	1.59 (5)	2.4278 (14)	170 (4)
C2—H2*A*⋯O2	0.98	2.57	3.2872 (17)	130
C2—H2*B*⋯Br2	0.98	3.09	4.0105 (14)	158
C2—H2*C*⋯I1^i^	0.98	3.18	4.0838 (14)	155
C3—H3*A*⋯Br2^ii^	0.98	3.07	3.8153 (14)	134
C3—H3*B*⋯O2^iii^	0.98	2.54	3.3481 (16)	140
C4—H4*A*⋯I1^i^	0.98	3.31	4.1081 (14)	140
C6—H6*A*⋯O1	0.98	2.64	3.3630 (16)	131
C6—H6*C*⋯Br1^ii^	0.98	3.06	3.7331 (14)	128
C7—H7*B*⋯Br1^iv^	0.98	2.97	3.8980 (15)	159
C8—H8*A*⋯Br1^v^	0.98	3.05	3.9722 (15)	157

Symmetry codes: (i) 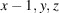 ; (ii) 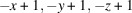 ; (iii) 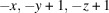 ; (iv) 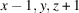 ; (v) 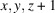 .

**Table 3:** Hydrogen-bond geometry (Å, °) for (III)

*D*—H⋯*A*	*D*—H	H⋯*A*	*D*⋯*A*	*D*—H⋯*A*
O1—H1⋯O1^i^	0.64 (3)	1.79 (3)	2.4261 (11)	170 (4)
C2—H2*A*⋯Cl1^ii^	0.98	2.93	3.7461 (8)	141
C2—H2*A*⋯O1^i^	0.98	2.61	3.3230 (9)	130
C2—H2*C*⋯Cl1	0.98	2.96	3.6902 (3)	132
C3—H3*A*⋯Cl1^iii^	0.98	2.95	3.6479 (9)	129
C3—H3*B*⋯Cl1^iv^	0.98	2.89	3.7897 (8)	153
C3—H3*C*⋯O1^v^	0.98	2.65	3.6256 (4)	176

Symmetry codes: (i) 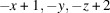 ; (ii) 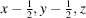 ; (iii) 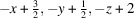 ; (iv) 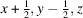 ; (v) 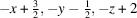 .

**Table 4:** Summary of short inter­atomic contacts (Å) in (**I**), (**II**) and (**III**)

Contact	Distance	Symmetry operation
(**I**)		
H1⋯O1	1.61	−*x* + 2, −*y* + 1, −*z* + 2
O1⋯H4*B*	2.73	*x* + 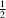 , −*y* + 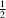 , *z* + 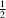
C2⋯H4*C*	3.06	−*x* + 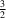 , *y* + 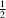 , −*z* + 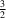
C2⋯H3*B*	3.09	*x* + 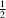 , −*y* + 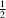 , *z* − 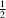
H2*A*⋯Br2	3.21	*x* + 1, *y*, *z*
H3*C*⋯Br2	3.05	*x*, *y*, *z*
H2*C*⋯Br1	3.13	−*x* + 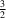 , *y* − 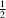 , −*z* + 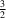
H3*A*⋯Br2	3.09	−*x* + 1, −*y* + 1, −*z* + 2
H4*C*⋯Br2	3.23	−*x* + 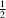 , *y* − 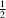 , −*z* + 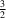
Br2⋯H2*B*	3.14	−*x* + 1, −*y* + 1, −*z* + 1
		
(**II**)		
H1⋯H2	0.86	*x*, *y*, *z*
C1⋯O1	3.24	−*x*, −*y* + 1, −*z* + 1
H3*C*⋯O1	2.68	−*x* + 1, −*y* + 1, −*z* + 1
H2*A*⋯H2*A*	2.54	−*x*, −*y*, −*z* + 1
H3*C*⋯Br2	3.23	*x*, *y* + 1, *z*
H2*B*⋯Br2	3.09	*x*, *y*, *z*
H2*C*⋯I1	3.18	*x* − 1, *y*, *z*
H3*A*⋯Br2	3.07	−*x* + 1, −*y* + 1, −*z* + 1
H3*C*⋯H6*A*	2.58	−*x* + 1, −*y* + 1, −*z* + 1
O2⋯H3*B*	2.54	−*x*, −*y* + 1, −*z* + 1
H8*B*⋯Br1	3.19	−*x* + 1, −*y*, −*z* + 1
H6*C*⋯Br1	3.06	−*x* + 1, −*y* + 1, −*z* + 1
H8*A*⋯Br1	3.05	*x*, *y*, *z* + 1
H7*B*⋯Br1	2.97	*x* − 1, *y*, *z* + 1
		
(**III**)		
H1⋯O1	1.79	−*x* + 1, *y*, −*z* + 2
H3*C*⋯O1	2.65	−*x* + 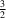 , *y* − 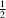 , −*z* + 2
H4*B*⋯Cl1	3.00	*x*, *y* − 1, *z*
H2*C*⋯Cl1	2.96	*x*, *y*, *z*
C3⋯C3	2.60	−*x* + 2, *y*, −*z* + 2
H3*A*⋯Cl1	2.95	−*x* + 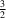 , *y* − 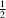 , −*z* + 2
H2*A*⋯Cl1	2.93	*x* − 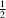 , *y* − 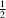 , *z*
H2*C*⋯H4*C*	2.58	*x* − 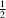 , *y* + 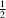 , *z*
H3*B*⋯Cl1	2.89	*x* + 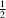 , *y* − 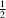 , *z*
I1⋯H4*A*	3.37	−*x* + 1, *y*, −*z* + 1
I1⋯H4*C*	3.36	−*x* + 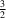 , *y* + 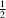 , −*z* + 1

**Table 5:** Percentage contributions of inter­atomic contacts to the Hirshfeld surface for (**I**), (**II**) and (**III**)

Contact	(**I**) (%)	(**II**) (%)	(**III**) (%)
H⋯H	57.5	60.3	88.9
Br⋯H/H⋯Br	24.0	15.2	–
O⋯H/H⋯O	13.3	12.0	6.5
C⋯H/H⋯C	3.0	2.7	2.0
Br⋯N/N⋯Br	1.0	–	–
N⋯H/H⋯N	0.9	2.4	0.8
Br⋯C/C⋯Br	0.5	–	–
I⋯H/H⋯I	–	4.7	–
O⋯C/C⋯O	–	2.2	–
O⋯N/N⋯O	–	0.3	–
O⋯O	–	0.1	–
Cl⋯N/N⋯Cl	–	–	0.8
Cl⋯C/C⋯Cl	–	–	0.7
Cl⋯H/H⋯Cl	–	–	0.4

**Table 6:** Experimental details For all structures: *Z* = 2. Experiments were carried out at 100 K with Mo *K*α radiation using a Bruker Kappa APEXII area-detector diffractometer. Absorption was corrected for by multi-scan methods (*SADABS*; Bruker, 2008 ▸).

	(**I**)	(**II**)	(**III**)
Crystal data			
Chemical formula	C_4_H_9_NO·C_4_H_10_NO^+^·Br_3_^−^	C_4_H_9_NO·C_4_H_10_NO^+^·Br_2_I^−^	C_4_H_9_NO·C_4_H_10_NO^+^·Cl_2_I^−^
*M*_r_	414.98	461.97	373.05
Crystal system, space group	Monoclinic, *P*2_1_/*n*	Triclinic, *P* 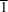	Monoclinic, *C*2/*m*
*a*, *b*, *c* (Å)	7.9009 (4), 10.3466 (6), 9.4948 (5)	7.2943 (3), 7.9544 (4), 13.6097 (7)	10.5264 (3), 6.7261 (2), 10.8124 (3)
α, β, γ (°)	90, 107.703 (2), 90	90.645 (2), 103.651 (2), 93.656 (2)	90, 105.950 (1), 90
*V* (Å^3^)	739.42 (7)	765.51 (6)	736.06 (4)
μ (mm^−1^)	8.17	7.30	2.53
Crystal size (mm)	0.24 × 0.20 × 0.14	0.14 × 0.08 × 0.06	0.20 × 0.18 × 0.14

Data collection			
*T*_min_, *T*_max_	0.315, 0.394	0.515, 0.669	0.630, 0.719
No. of measured, independent and observed [*I* > 2σ(*I*)] reflections	11995, 3239, 2402	30601, 6766, 5446	13071, 1745, 1745
*R*_int_	0.026	0.021	0.014
(sin θ/λ)_max_ (Å^−1^)	0.807	0.811	0.811

Refinement			
*R*[*F* ^2^ > 2σ(*F* ^2^)], *wR*(*F* ^2^), *S*	0.025, 0.056, 1.01	0.019, 0.038, 1.03	0.008, 0.022, 1.06
No. of reflections	3239	6766	1745
No. of parameters	73	149	52
H-atom treatment	H-atom parameters constrained	H atoms treated by a mixture of independent and constrained refinement	H atoms treated by a mixture of independent and constrained refinement
Δρ_max_, Δρ_min_ (e Å^−3^)	0.43, −0.78	0.52, −0.62	0.46, −0.26

Computer programs: *APEX2* and *SAINT* (Bruker, 2013 ▸), *SHELXT* (Sheldrick, 2015*a* ▸), *SHELXL* (Sheldrick, 2015*b* ▸), *ORTEP-3 for Windows* (Farrugia, 2012 ▸) and *PLATON* (Spek, 2020 ▸).

## supplementary crystallographic information

### N,N-Dimethylacetamide–1-(dimethyl-λ4-azanylidene)ethan-1-ol tribromide (1/1) (I) Crystal data

**Table:**

C_4_H_9_NO·C_4_H_10_NO^+^·Br_3_^−^	*F*(000) = 404
*M**_r_* = 414.98	*D*_x_ = 1.864 Mg m^−^^3^
Monoclinic, *P*2_1_/*n*	Mo *K*α radiation, λ = 0.71073 Å
*a* = 7.9009 (4) Å	Cell parameters from 3187 reflections
*b* = 10.3466 (6) Å	θ = 3.0–34.7°
*c* = 9.4948 (5) Å	µ = 8.17 mm^−^^1^
β = 107.703 (2)°	*T* = 100 K
*V* = 739.42 (7) Å^3^	Fragment, orange
*Z* = 2	0.24 × 0.20 × 0.14 mm

### N,N-Dimethylacetamide–1-(dimethyl-λ4-azanylidene)ethan-1-ol tribromide (1/1) (I) Data collection

**Table:**

Bruker Kappa APEXII area-detector diffractometer	2402 reflections with *I* > 2σ(*I*)
ω– and φ–scans	*R*_int_ = 0.026
Absorption correction: multi-scan (SADABS; Bruker, 2008)	θ_max_ = 35.0°, θ_min_ = 4.5°
*T*_min_ = 0.315, *T*_max_ = 0.394	*h* = −12→12
11995 measured reflections	*k* = −16→16
3239 independent reflections	*l* = −15→15

### N,N-Dimethylacetamide–1-(dimethyl-λ4-azanylidene)ethan-1-ol tribromide (1/1) (I) Refinement

**Table:**

Refinement on *F*^2^	Primary atom site location: dual
Least-squares matrix: full	Hydrogen site location: mixed
*R*[*F*^2^ > 2σ(*F*^2^)] = 0.025	H-atom parameters constrained
*wR*(*F*^2^) = 0.056	*w* = 1/[σ^2^(*F*_o_^2^) + (0.0249*P*)^2^ + 0.0334*P*] where *P* = (*F*_o_^2^ + 2*F*_c_^2^)/3
*S* = 1.01	(Δ/σ)_max_ = 0.002
3239 reflections	Δρ_max_ = 0.43 e Å^−^^3^
73 parameters	Δρ_min_ = −0.78 e Å^−^^3^
0 restraints	

### N,N-Dimethylacetamide–1-(dimethyl-λ4-azanylidene)ethan-1-ol tribromide (1/1) (I) Special details

**Table:**

Geometry. All esds (except the esd in the dihedral angle between two l.s. planes) are estimated using the full covariance matrix. The cell esds are taken into account individually in the estimation of esds in distances, angles and torsion angles; correlations between esds in cell parameters are only used when they are defined by crystal symmetry. An approximate (isotropic) treatment of cell esds is used for estimating esds involving l.s. planes.

### N,N-Dimethylacetamide–1-(dimethyl-λ4-azanylidene)ethan-1-ol tribromide (1/1) (I) Fractional atomic coordinates and isotropic or equivalent isotropic displacement parameters (Å^2^)

**Table:**

	*x*	*y*	*z*	*U*_iso_*/*U*_eq_	
Br1	0.500000	0.500000	0.500000	0.01461 (5)	
Br2	0.26135 (2)	0.50973 (2)	0.62805 (2)	0.02228 (5)	
O1	0.85472 (13)	0.45244 (10)	0.98120 (11)	0.0190 (2)	
H1	1.000000	0.500000	1.000000	0.029*	
N1	0.66034 (15)	0.29961 (10)	0.87188 (13)	0.0153 (2)	
C1	0.80749 (18)	0.36364 (12)	0.88341 (15)	0.0146 (2)	
C2	0.91855 (19)	0.33325 (14)	0.78479 (16)	0.0193 (3)	
H2A	1.028724	0.383619	0.816158	0.029*	
H2B	0.852428	0.355410	0.682486	0.029*	
H2C	0.947123	0.240846	0.791228	0.029*	
C3	0.55467 (19)	0.32410 (14)	0.97137 (17)	0.0198 (3)	
H3A	0.624789	0.374837	1.056242	0.030*	
H3B	0.521000	0.241642	1.006057	0.030*	
H3C	0.447312	0.372248	0.918561	0.030*	
C4	0.5868 (2)	0.20268 (14)	0.75764 (17)	0.0214 (3)	
H4A	0.658131	0.199738	0.689389	0.032*	
H4B	0.464005	0.225520	0.703018	0.032*	
H4C	0.589038	0.117778	0.803936	0.032*	

### N,N-Dimethylacetamide–1-(dimethyl-λ4-azanylidene)ethan-1-ol tribromide (1/1) (I) Atomic displacement parameters (Å^2^)

**Table:**

	*U*^11^	*U*^22^	*U*^33^	*U*^12^	*U*^13^	*U*^23^
Br1	0.01420 (9)	0.01516 (8)	0.01360 (8)	0.00133 (6)	0.00293 (7)	−0.00072 (6)
Br2	0.01914 (8)	0.02903 (8)	0.02132 (8)	0.00196 (6)	0.01013 (6)	−0.00041 (6)
O1	0.0217 (5)	0.0179 (4)	0.0149 (5)	−0.0066 (4)	0.0018 (4)	−0.0020 (4)
N1	0.0183 (6)	0.0141 (5)	0.0135 (5)	−0.0035 (4)	0.0050 (5)	−0.0023 (4)
C1	0.0168 (6)	0.0132 (5)	0.0116 (6)	0.0002 (5)	0.0009 (5)	0.0037 (4)
C2	0.0210 (7)	0.0196 (6)	0.0184 (7)	−0.0002 (5)	0.0078 (6)	0.0016 (5)
C3	0.0205 (7)	0.0214 (6)	0.0200 (7)	−0.0025 (5)	0.0098 (6)	−0.0021 (5)
C4	0.0255 (7)	0.0186 (6)	0.0191 (7)	−0.0073 (5)	0.0056 (6)	−0.0063 (5)

### N,N-Dimethylacetamide–1-(dimethyl-λ4-azanylidene)ethan-1-ol tribromide (1/1) (I) Geometric parameters (Å, º)

**Table:**

Br1—Br2^i^	2.5372 (2)	C2—H2B	0.9800
Br1—Br2	2.5372 (2)	C2—H2C	0.9800
O1—C1	1.2786 (16)	C3—H3A	0.9800
O1—H1	1.2112	C3—H3B	0.9800
N1—C1	1.3134 (17)	C3—H3C	0.9800
N1—C3	1.4605 (18)	C4—H4A	0.9800
N1—C4	1.4618 (18)	C4—H4B	0.9800
C1—C2	1.4984 (19)	C4—H4C	0.9800
C2—H2A	0.9800		
			
Br2^i^—Br1—Br2	180.0	H2B—C2—H2C	109.5
C1—O1—H1	116.95	N1—C3—H3A	109.5
C1—N1—C3	121.62 (11)	N1—C3—H3B	109.5
C1—N1—C4	123.36 (12)	H3A—C3—H3B	109.5
C3—N1—C4	115.00 (11)	N1—C3—H3C	109.5
O1—C1—N1	118.58 (12)	H3A—C3—H3C	109.5
O1—C1—C2	120.50 (12)	H3B—C3—H3C	109.5
N1—C1—C2	120.92 (12)	N1—C4—H4A	109.5
C1—C2—H2A	109.5	N1—C4—H4B	109.5
C1—C2—H2B	109.5	H4A—C4—H4B	109.5
H2A—C2—H2B	109.5	N1—C4—H4C	109.5
C1—C2—H2C	109.5	H4A—C4—H4C	109.5
H2A—C2—H2C	109.5	H4B—C4—H4C	109.5
			
C3—N1—C1—O1	−2.46 (19)	C3—N1—C1—C2	177.27 (12)
C4—N1—C1—O1	175.52 (13)	C4—N1—C1—C2	−4.8 (2)

Symmetry code: (i) −*x*+1, −*y*+1, −*z*+1.

### N,N-Dimethylacetamide–1-(dimethyl-λ4-azanylidene)ethan-1-ol tribromide (1/1) (I) Hydrogen-bond geometry (Å, º)

**Table:**

*D*—H···*A*	*D*—H	H···*A*	*D*···*A*	*D*—H···*A*
C2—H2*A*···O1^ii^	0.98	2.52	3.2622 (18)	132
C2—H2*B*···Br2^i^	0.98	3.14	4.0788 (15)	162
C2—H2*C*···Br1^iii^	0.98	3.13	3.9596 (14)	143
C3—H3*A*···Br2^iv^	0.98	3.10	4.0216 (15)	158
C3—H3*C*···Br2	0.98	3.05	3.8847 (15)	143
O1—H1···O1^ii^	1.21	1.21	2.4224 (15)	180
C3—H3*A*···O1	0.98	2.29	2.6940 (19)	104

Symmetry codes: (i) −*x*+1, −*y*+1, −*z*+1; (ii) −*x*+2, −*y*+1, −*z*+2; (iii) −*x*+3/2, *y*−1/2, −*z*+3/2; (iv) −*x*+1, −*y*+1, −*z*+2.

### N,N-Dimethylacetamide–1-(dimethyl-λ4-azanylidene)ethan-1-ol dibromidoiodate (1/1) (II) Crystal data

**Table:**

C_4_H_9_NO·C_4_H_10_NO^−^·Br_2_I^−^	*Z* = 2
*M**_r_* = 461.97	*F*(000) = 440
Triclinic, *P*1	*D*_x_ = 2.004 Mg m^−^^3^
*a* = 7.2943 (3) Å	Mo *K*α radiation, λ = 0.71073 Å
*b* = 7.9544 (4) Å	Cell parameters from 9984 reflections
*c* = 13.6097 (7) Å	θ = 2.9–35.2°
α = 90.645 (2)°	µ = 7.30 mm^−^^1^
β = 103.651 (2)°	*T* = 100 K
γ = 93.656 (2)°	Fragment, orange
*V* = 765.51 (6) Å^3^	0.14 × 0.08 × 0.06 mm

### N,N-Dimethylacetamide–1-(dimethyl-λ4-azanylidene)ethan-1-ol dibromidoiodate (1/1) (II) Data collection

**Table:**

Bruker Kappa APEXII area-detector diffractometer	5446 reflections with *I* > 2σ(*I*)
ω– and φ–scans	*R*_int_ = 0.021
Absorption correction: multi-scan (SADABS; Bruker, 2008)	θ_max_ = 35.2°, θ_min_ = 4.3°
*T*_min_ = 0.515, *T*_max_ = 0.669	*h* = −11→11
30601 measured reflections	*k* = −12→12
6766 independent reflections	*l* = −22→21

### N,N-Dimethylacetamide–1-(dimethyl-λ4-azanylidene)ethan-1-ol dibromidoiodate (1/1) (II) Refinement

**Table:**

Refinement on *F*^2^	Primary atom site location: dual
Least-squares matrix: full	Hydrogen site location: mixed
*R*[*F*^2^ > 2σ(*F*^2^)] = 0.019	H atoms treated by a mixture of independent and constrained refinement
*wR*(*F*^2^) = 0.038	*w* = 1/[σ^2^(*F*_o_^2^) + (0.0119*P*)^2^ + 0.2374*P*] where *P* = (*F*_o_^2^ + 2*F*_c_^2^)/3
*S* = 1.03	(Δ/σ)_max_ = 0.001
6766 reflections	Δρ_max_ = 0.52 e Å^−^^3^
149 parameters	Δρ_min_ = −0.62 e Å^−^^3^
0 restraints	

### N,N-Dimethylacetamide–1-(dimethyl-λ4-azanylidene)ethan-1-ol dibromidoiodate (1/1) (II) Special details

**Table:**

Geometry. All esds (except the esd in the dihedral angle between two l.s. planes) are estimated using the full covariance matrix. The cell esds are taken into account individually in the estimation of esds in distances, angles and torsion angles; correlations between esds in cell parameters are only used when they are defined by crystal symmetry. An approximate (isotropic) treatment of cell esds is used for estimating esds involving l.s. planes.

### N,N-Dimethylacetamide–1-(dimethyl-λ4-azanylidene)ethan-1-ol dibromidoiodate (1/1) (II) Fractional atomic coordinates and isotropic or equivalent isotropic displacement parameters (Å^2^)

**Table:**

	*x*	*y*	*z*	*U*_iso_*/*U*_eq_	Occ. (<1)
I1	0.65027 (2)	0.18015 (2)	0.21358 (2)	0.01710 (2)	
Br1	0.80118 (2)	0.33097 (2)	0.06912 (2)	0.02346 (3)	
Br2	0.51412 (2)	0.02963 (2)	0.35979 (2)	0.02157 (3)	
O1	0.20994 (14)	0.43799 (12)	0.57436 (7)	0.01964 (19)	
H1	0.187 (6)	0.388 (6)	0.617 (3)	0.029*	0.49 (4)
O2	0.10583 (13)	0.27819 (13)	0.70454 (7)	0.02121 (19)	
H2	0.151 (5)	0.325 (6)	0.659 (3)	0.032*	0.51 (4)
N1	0.22672 (15)	0.45877 (13)	0.41323 (8)	0.01563 (19)	
N2	0.14789 (15)	0.19762 (14)	0.86403 (8)	0.0168 (2)	
C1	0.17805 (16)	0.37110 (15)	0.48569 (9)	0.0147 (2)	
C2	0.08734 (19)	0.19621 (16)	0.46499 (10)	0.0204 (2)	
H2A	0.080500	0.143984	0.529020	0.031*	
H2B	0.162652	0.129403	0.430349	0.031*	
H2C	−0.040553	0.200696	0.422060	0.031*	
C3	0.31786 (18)	0.62905 (16)	0.43360 (10)	0.0198 (2)	
H3A	0.354648	0.651700	0.506798	0.030*	
H3B	0.229380	0.711180	0.401612	0.030*	
H3C	0.430563	0.638270	0.406042	0.030*	
C4	0.1909 (2)	0.39725 (18)	0.30812 (10)	0.0215 (3)	
H4A	0.104320	0.295852	0.298507	0.032*	
H4B	0.310488	0.370162	0.292695	0.032*	
H4C	0.134030	0.484580	0.262824	0.032*	
C5	0.21588 (17)	0.26890 (15)	0.79243 (9)	0.0155 (2)	
C6	0.41829 (18)	0.33486 (16)	0.81159 (10)	0.0192 (2)	
H6A	0.438604	0.396328	0.752644	0.029*	
H6B	0.500151	0.240498	0.823484	0.029*	
H6C	0.448650	0.410935	0.871198	0.029*	
C7	−0.0468 (2)	0.12246 (19)	0.84285 (11)	0.0243 (3)	
H7A	−0.107738	0.135459	0.771288	0.036*	
H7B	−0.117410	0.179306	0.884927	0.036*	
H7C	−0.045456	0.002396	0.858179	0.036*	
C8	0.2581 (2)	0.18213 (19)	0.96814 (10)	0.0236 (3)	
H8A	0.382190	0.242901	0.976432	0.035*	
H8B	0.274782	0.062886	0.982492	0.035*	
H8C	0.191026	0.230260	1.015097	0.035*	

### N,N-Dimethylacetamide–1-(dimethyl-λ4-azanylidene)ethan-1-ol dibromidoiodate (1/1) (II) Atomic displacement parameters (Å^2^)

**Table:**

	*U*^11^	*U*^22^	*U*^33^	*U*^12^	*U*^13^	*U*^23^
I1	0.01763 (4)	0.01708 (3)	0.01549 (4)	0.00366 (3)	0.00121 (3)	−0.00183 (3)
Br1	0.02527 (7)	0.02855 (7)	0.01748 (7)	0.00332 (5)	0.00655 (5)	−0.00017 (5)
Br2	0.02328 (7)	0.01921 (6)	0.02211 (7)	0.00054 (5)	0.00536 (5)	0.00173 (5)
O1	0.0235 (5)	0.0221 (4)	0.0133 (4)	0.0003 (4)	0.0045 (4)	0.0014 (3)
O2	0.0171 (4)	0.0323 (5)	0.0145 (4)	0.0026 (4)	0.0038 (4)	0.0061 (4)
N1	0.0139 (5)	0.0185 (4)	0.0143 (5)	0.0007 (4)	0.0031 (4)	0.0021 (4)
N2	0.0175 (5)	0.0207 (5)	0.0125 (5)	0.0015 (4)	0.0040 (4)	0.0017 (4)
C1	0.0113 (5)	0.0178 (5)	0.0146 (5)	0.0026 (4)	0.0020 (4)	0.0022 (4)
C2	0.0220 (6)	0.0181 (5)	0.0202 (6)	−0.0019 (5)	0.0039 (5)	0.0016 (5)
C3	0.0168 (6)	0.0189 (5)	0.0227 (6)	−0.0016 (4)	0.0030 (5)	0.0038 (5)
C4	0.0237 (7)	0.0266 (6)	0.0148 (6)	0.0022 (5)	0.0059 (5)	0.0006 (5)
C5	0.0164 (5)	0.0161 (5)	0.0149 (5)	0.0043 (4)	0.0047 (4)	0.0006 (4)
C6	0.0182 (6)	0.0213 (5)	0.0180 (6)	0.0003 (4)	0.0043 (5)	0.0013 (5)
C7	0.0194 (6)	0.0337 (7)	0.0208 (7)	−0.0019 (5)	0.0075 (5)	0.0031 (5)
C8	0.0270 (7)	0.0292 (7)	0.0133 (6)	0.0001 (5)	0.0027 (5)	0.0039 (5)

### N,N-Dimethylacetamide–1-(dimethyl-λ4-azanylidene)ethan-1-ol dibromidoiodate (1/1) (II) Geometric parameters (Å, º)

**Table:**

I1—Br2	2.6860 (2)	C3—H3A	0.9800
I1—Br1	2.7243 (2)	C3—H3B	0.9800
O1—C1	1.2771 (15)	C3—H3C	0.9800
O1—H1	0.75 (5)	C4—H4A	0.9800
O2—C5	1.2794 (15)	C4—H4B	0.9800
O2—H2	0.85 (5)	C4—H4C	0.9800
N1—C1	1.3168 (16)	C5—C6	1.4965 (18)
N1—C3	1.4640 (16)	C6—H6A	0.9800
N1—C4	1.4648 (17)	C6—H6B	0.9800
N2—C5	1.3121 (16)	C6—H6C	0.9800
N2—C8	1.4656 (17)	C7—H7A	0.9800
N2—C7	1.4673 (17)	C7—H7B	0.9800
C1—C2	1.4961 (17)	C7—H7C	0.9800
C2—H2A	0.9800	C8—H8A	0.9800
C2—H2B	0.9800	C8—H8B	0.9800
C2—H2C	0.9800	C8—H8C	0.9800
			
Br2—I1—Br1	177.942 (6)	H4A—C4—H4B	109.5
C1—O1—H1	120 (3)	N1—C4—H4C	109.5
C5—O2—H2	118 (3)	H4A—C4—H4C	109.5
C1—N1—C3	120.94 (11)	H4B—C4—H4C	109.5
C1—N1—C4	123.49 (11)	O2—C5—N2	118.46 (12)
C3—N1—C4	115.55 (10)	O2—C5—C6	120.28 (11)
C5—N2—C8	123.75 (11)	N2—C5—C6	121.25 (11)
C5—N2—C7	120.94 (11)	C5—C6—H6A	109.5
C8—N2—C7	115.28 (11)	C5—C6—H6B	109.5
O1—C1—N1	118.77 (11)	H6A—C6—H6B	109.5
O1—C1—C2	120.38 (11)	C5—C6—H6C	109.5
N1—C1—C2	120.84 (11)	H6A—C6—H6C	109.5
C1—C2—H2A	109.5	H6B—C6—H6C	109.5
C1—C2—H2B	109.5	N2—C7—H7A	109.5
H2A—C2—H2B	109.5	N2—C7—H7B	109.5
C1—C2—H2C	109.5	H7A—C7—H7B	109.5
H2A—C2—H2C	109.5	N2—C7—H7C	109.5
H2B—C2—H2C	109.5	H7A—C7—H7C	109.5
N1—C3—H3A	109.5	H7B—C7—H7C	109.5
N1—C3—H3B	109.5	N2—C8—H8A	109.5
H3A—C3—H3B	109.5	N2—C8—H8B	109.5
N1—C3—H3C	109.5	H8A—C8—H8B	109.5
H3A—C3—H3C	109.5	N2—C8—H8C	109.5
H3B—C3—H3C	109.5	H8A—C8—H8C	109.5
N1—C4—H4A	109.5	H8B—C8—H8C	109.5
N1—C4—H4B	109.5		
			
C3—N1—C1—O1	0.75 (17)	C8—N2—C5—O2	−178.76 (12)
C4—N1—C1—O1	−177.75 (11)	C7—N2—C5—O2	3.09 (18)
C3—N1—C1—C2	−179.15 (11)	C8—N2—C5—C6	2.56 (19)
C4—N1—C1—C2	2.35 (18)	C7—N2—C5—C6	−175.59 (12)

### N,N-Dimethylacetamide–1-(dimethyl-λ4-azanylidene)ethan-1-ol dibromidoiodate (1/1) (II) Hydrogen-bond geometry (Å, º)

**Table:**

*D*—H···*A*	*D*—H	H···*A*	*D*···*A*	*D*—H···*A*
O1—H1···O2	0.75 (5)	1.69 (5)	2.4278 (13)	173 (4)
O2—H2···O1	0.85 (5)	1.59 (5)	2.4278 (14)	170 (4)
C2—H2*A*···O2	0.98	2.57	3.2872 (17)	130
C2—H2*B*···Br2	0.98	3.09	4.0105 (14)	158
C2—H2*C*···I1^i^	0.98	3.18	4.0838 (14)	155
C3—H3*A*···Br2^ii^	0.98	3.07	3.8153 (14)	134
C3—H3*B*···O2^iii^	0.98	2.54	3.3481 (16)	140
C4—H4*A*···I1^i^	0.98	3.31	4.1081 (14)	140
C6—H6*A*···O1	0.98	2.64	3.3630 (16)	131
C6—H6*C*···Br1^ii^	0.98	3.06	3.7331 (14)	128
C7—H7*B*···Br1^iv^	0.98	2.97	3.8980 (15)	159
C8—H8*A*···Br1^v^	0.98	3.05	3.9722 (15)	157
C3—H3*A*···O1	0.98	2.26	2.6878 (16)	105
C7—H7*A*···O2	0.98	2.24	2.6801 (18)	106

Symmetry codes: (i) *x*−1, *y*, *z*; (ii) −*x*+1, −*y*+1, −*z*+1; (iii) −*x*, −*y*+1, −*z*+1; (iv) *x*−1, *y*, *z*+1; (v) *x*, *y*, *z*+1.

### N,N-Dimethylacetamide–1-(dimethyl-λ4-azanylidene)ethan-1-ol dichloridoiodate (1/1) (III) Crystal data

**Table:**

C_4_H_9_NO·C_4_H_10_NO^−^·Cl_2_I^−^	*F*(000) = 368
*M**_r_* = 373.05	*D*_x_ = 1.683 Mg m^−^^3^
Monoclinic, *C*2/*m*	Mo *K*α radiation, λ = 0.71073 Å
*a* = 10.5264 (3) Å	Cell parameters from 9986 reflections
*b* = 6.7261 (2) Å	θ = 3.6–35.1°
*c* = 10.8124 (3) Å	µ = 2.53 mm^−^^1^
β = 105.950 (1)°	*T* = 100 K
*V* = 736.06 (4) Å^3^	Fragment, orange
*Z* = 2	0.20 × 0.18 × 0.14 mm

### N,N-Dimethylacetamide–1-(dimethyl-λ4-azanylidene)ethan-1-ol dichloridoiodate (1/1) (III) Data collection

**Table:**

Bruker Kappa APEXII area-detector diffractometer	1745 reflections with *I* > 2σ(*I*)
ω– and φ–scans	*R*_int_ = 0.014
Absorption correction: multi-scan (SADABS; Bruker, 2008)	θ_max_ = 35.2°, θ_min_ = 4.4°
*T*_min_ = 0.630, *T*_max_ = 0.719	*h* = −16→16
13071 measured reflections	*k* = −10→10
1745 independent reflections	*l* = −17→16

### N,N-Dimethylacetamide–1-(dimethyl-λ4-azanylidene)ethan-1-ol dichloridoiodate (1/1) (III) Refinement

**Table:**

Refinement on *F*^2^	Primary atom site location: dual
Least-squares matrix: full	Hydrogen site location: mixed
*R*[*F*^2^ > 2σ(*F*^2^)] = 0.008	H atoms treated by a mixture of independent and constrained refinement
*wR*(*F*^2^) = 0.022	*w* = 1/[σ^2^(*F*_o_^2^) + (0.0153*P*)^2^ + 0.0381*P*] where *P* = (*F*_o_^2^ + 2*F*_c_^2^)/3
*S* = 1.06	(Δ/σ)_max_ = 0.003
1745 reflections	Δρ_max_ = 0.46 e Å^−^^3^
52 parameters	Δρ_min_ = −0.25 e Å^−^^3^
0 restraints	

### N,N-Dimethylacetamide–1-(dimethyl-λ4-azanylidene)ethan-1-ol dichloridoiodate (1/1) (III) Special details

**Table:**

Geometry. All esds (except the esd in the dihedral angle between two l.s. planes) are estimated using the full covariance matrix. The cell esds are taken into account individually in the estimation of esds in distances, angles and torsion angles; correlations between esds in cell parameters are only used when they are defined by crystal symmetry. An approximate (isotropic) treatment of cell esds is used for estimating esds involving l.s. planes.

### N,N-Dimethylacetamide–1-(dimethyl-λ4-azanylidene)ethan-1-ol dichloridoiodate (1/1) (III) Fractional atomic coordinates and isotropic or equivalent isotropic displacement parameters (Å^2^)

**Table:**

	*x*	*y*	*z*	*U*_iso_*/*U*_eq_	Occ. (<1)
I1	0.500000	0.500000	0.500000	0.01617 (2)	
Cl1	0.60222 (2)	0.500000	0.74187 (2)	0.02093 (3)	
O1	0.60066 (6)	0.000000	0.96603 (6)	0.02505 (10)	
H1	0.543 (3)	0.000000	0.977 (3)	0.038*	0.5
N1	0.70175 (6)	0.000000	0.81090 (6)	0.01741 (9)	
C1	0.59151 (7)	0.000000	0.84597 (6)	0.01742 (10)	
C2	0.45979 (7)	0.000000	0.74807 (8)	0.02291 (12)	
H2A	0.390193	−0.021971	0.790959	0.034*	0.5
H2B	0.457166	−0.106447	0.685527	0.034*	0.5
H2C	0.445707	0.128418	0.703565	0.034*	0.5
C3	0.82909 (8)	0.000000	0.90856 (8)	0.02547 (13)	
H3A	0.824028	0.085866	0.980273	0.038*	0.5
H3B	0.897594	0.049926	0.870861	0.038*	0.5
H3C	0.851076	−0.135792	0.940046	0.038*	0.5
C4	0.70493 (8)	0.000000	0.67698 (7)	0.02212 (12)	
H4A	0.622560	0.056849	0.623015	0.033*	0.5
H4B	0.714477	−0.136724	0.649508	0.033*	0.5
H4C	0.779897	0.079874	0.668365	0.033*	0.5

### N,N-Dimethylacetamide–1-(dimethyl-λ4-azanylidene)ethan-1-ol dichloridoiodate (1/1) (III) Atomic displacement parameters (Å^2^)

**Table:**

	*U*^11^	*U*^22^	*U*^33^	*U*^12^	*U*^13^	*U*^23^
I1	0.01526 (3)	0.01688 (3)	0.01778 (3)	0.000	0.00693 (2)	0.000
Cl1	0.02127 (7)	0.02325 (7)	0.01802 (6)	0.000	0.00499 (5)	0.000
O1	0.0226 (2)	0.0357 (3)	0.0204 (2)	0.000	0.01195 (19)	0.000
N1	0.0170 (2)	0.0189 (2)	0.0187 (2)	0.000	0.00881 (18)	0.000
C1	0.0173 (2)	0.0164 (2)	0.0208 (2)	0.000	0.0090 (2)	0.000
C2	0.0176 (3)	0.0254 (3)	0.0260 (3)	0.000	0.0065 (2)	0.000
C3	0.0172 (3)	0.0356 (4)	0.0242 (3)	0.000	0.0067 (2)	0.000
C4	0.0235 (3)	0.0260 (3)	0.0201 (3)	0.000	0.0113 (2)	0.000

### N,N-Dimethylacetamide–1-(dimethyl-λ4-azanylidene)ethan-1-ol dichloridoiodate (1/1) (III) Geometric parameters (Å, º)

**Table:**

I1—Cl1	2.5398 (2)	C2—H2C^ii^	0.980 (10)
I1—Cl1^i^	2.5398 (2)	C3—H3A	0.9800
O1—C1	1.2750 (8)	C3—H3B	0.9800
O1—H1	0.64 (3)	C3—H3C	0.9800
N1—C1	1.3161 (8)	C3—H3A^ii^	0.980 (9)
N1—C4	1.4576 (9)	C3—H3B^ii^	0.980 (6)
N1—C3	1.4608 (10)	C3—H3C^ii^	0.980 (3)
C1—C2	1.4955 (10)	C4—H4A	0.9800
C2—H2A	0.9800	C4—H4B	0.9800
C2—H2B	0.9800	C4—H4C	0.9800
C2—H2C	0.9800	C4—H4A^ii^	0.980 (7)
C2—H2A^ii^	0.980 (5)	C4—H4B^ii^	0.9800 (17)
C2—H2B^ii^	0.980 (15)	C4—H4C^ii^	0.980 (8)
			
Cl1—I1—Cl1^i^	180.0	H3A—C3—H3A^ii^	72.2
C1—O1—H1	112 (3)	H3B—C3—H3A^ii^	137.5
C1—N1—C4	123.30 (6)	H3C—C3—H3A^ii^	40.1
C1—N1—C3	119.89 (6)	N1—C3—H3B^ii^	109.47 (14)
C4—N1—C3	116.81 (6)	H3A—C3—H3B^ii^	137.5
O1—C1—N1	117.87 (7)	H3B—C3—H3B^ii^	40.1
O1—C1—C2	121.10 (6)	H3C—C3—H3B^ii^	72.2
N1—C1—C2	121.02 (6)	H3A^ii^—C3—H3B^ii^	109.5
C1—C2—H2A	109.5	N1—C3—H3C^ii^	109.47 (6)
C1—C2—H2B	109.5	H3A—C3—H3C^ii^	40.1
H2A—C2—H2B	109.5	H3B—C3—H3C^ii^	72.2
C1—C2—H2C	109.5	H3C—C3—H3C^ii^	137.5
H2A—C2—H2C	109.5	H3A^ii^—C3—H3C^ii^	109.5
H2B—C2—H2C	109.5	H3B^ii^—C3—H3C^ii^	109.5
C1—C2—H2A^ii^	109.47 (11)	N1—C4—H4A	109.5
H2B—C2—H2A^ii^	123.6	N1—C4—H4B	109.5
H2C—C2—H2A^ii^	93.9	H4A—C4—H4B	109.5
C1—C2—H2B^ii^	109.5 (3)	N1—C4—H4C	109.5
H2A—C2—H2B^ii^	123.6	H4A—C4—H4C	109.5
H2B—C2—H2B^ii^	93.9	H4B—C4—H4C	109.5
H2C—C2—H2B^ii^	17.3	N1—C4—H4A^ii^	109.47 (15)
H2A^ii^—C2—H2B^ii^	109.5	H4A—C4—H4A^ii^	45.9
C1—C2—H2C^ii^	109.5 (2)	H4B—C4—H4A^ii^	66.5
H2A—C2—H2C^ii^	93.9	H4C—C4—H4A^ii^	139.6
H2B—C2—H2C^ii^	17.3	N1—C4—H4B^ii^	109.47 (3)
H2C—C2—H2C^ii^	123.6	H4A—C4—H4B^ii^	66.5
H2A^ii^—C2—H2C^ii^	109.5	H4B—C4—H4B^ii^	139.6
H2B^ii^—C2—H2C^ii^	109.5	H4C—C4—H4B^ii^	45.9
N1—C3—H3A	109.5	H4A^ii^—C4—H4B^ii^	109.5
N1—C3—H3B	109.5	N1—C4—H4C^ii^	109.47 (18)
H3A—C3—H3B	109.5	H4A—C4—H4C^ii^	139.6
N1—C3—H3C	109.5	H4B—C4—H4C^ii^	45.9
H3A—C3—H3C	109.5	H4C—C4—H4C^ii^	66.5
H3B—C3—H3C	109.5	H4A^ii^—C4—H4C^ii^	109.5
N1—C3—H3A^ii^	109.5 (2)	H4B^ii^—C4—H4C^ii^	109.5
			
C4—N1—C1—O1	180.000 (1)	C4—N1—C1—C2	0.000 (1)
C3—N1—C1—O1	0.000 (1)	C3—N1—C1—C2	180.000 (1)

Symmetry codes: (i) −*x*+1, −*y*+1, −*z*+1; (ii) *x*, −*y*, *z*.

### N,N-Dimethylacetamide–1-(dimethyl-λ4-azanylidene)ethan-1-ol dichloridoiodate (1/1) (III) Hydrogen-bond geometry (Å, º)

**Table:**

*D*—H···*A*	*D*—H	H···*A*	*D*···*A*	*D*—H···*A*
O1—H1···O1^iii^	0.64 (3)	1.79 (3)	2.4261 (11)	170 (4)
C2—H2*A*···Cl1^iv^	0.98	2.93	3.7461 (8)	141
C2—H2*A*···O1^iii^	0.98	2.61	3.3230 (9)	130
C2—H2*C*···Cl1	0.98	2.96	3.6902 (3)	132
C3—H3*A*···Cl1^v^	0.98	2.95	3.6479 (9)	129
C3—H3*B*···Cl1^vi^	0.98	2.89	3.7897 (8)	153
C3—H3*C*···O1^vii^	0.98	2.65	3.6256 (4)	176

Symmetry codes: (iii) −*x*+1, −*y*, −*z*+2; (iv) *x*−1/2, *y*−1/2, *z*; (v) −*x*+3/2, −*y*+1/2, −*z*+2; (vi) *x*+1/2, *y*−1/2, *z*; (vii) −*x*+3/2, −*y*−1/2, −*z*+2.
